# Gemfibrozil mitigates caspase-11-driven myocardial pyroptosis in ischemia/reperfusion injury in mice

**DOI:** 10.1016/j.jmccpl.2025.100292

**Published:** 2025-03-10

**Authors:** Tetsuro Marunouchi, Mayu Kyono, Naoko Kikuchi, Kouichi Tanonaka

**Affiliations:** Department of Molecular and Cellular Pharmacology, Tokyo University of Pharmacy and Life Sciences, Japan

**Keywords:** Caspase-11, Pyroptosis, Gemfibrozil, Ischemia, Reperfusion

## Abstract

The size of the infarct area following acute myocardial infarction (AMI) is a critical prognostic factor. Caspase-11-dependent pyroptosis has been implicated as a key mechanism driving cardiomyocyte death after AMI. However, no therapeutic agents have been developed to inhibit myocardial cell death by targeting caspase-11. This study investigates the effects of gemfibrozil, a potential caspase-11 inhibitor, on ischemia/reperfusion-induced myocardial pyroptosis in mice. To model AMI, the left coronary artery of C57BL/6 N mice was ligated for 1 h, followed by reperfusion. Levels of cleaved caspase-11 and the N-terminal fragment of gasdermin D (GSDMD-N) in ischemic myocardial tissue increased progressively over time after ischemia/reperfusion. Gemfibrozil treatment during reperfusion significantly attenuated these increases in cleaved caspase-11 and GSDMD-N levels. Moreover, gemfibrozil reduced the extent of myocardial infarct size during reperfusion. In cultured cardiomyocytes isolated from adult mice, hypoxia/reoxygenation-induced increases in caspase-11 and GSDMD cleavage were similarly mitigated by gemfibrozil, which concurrently prevented necrotic cell death. These findings demonstrate the involvement of caspase-11-dependent pyroptosis in myocardial cell death following ischemia/reperfusion and suggest that gemfibrozil holds promise as a therapeutic agent for reducing myocardial infarct size after AMI.

## Highlights

• Caspase-11 pyroptosis is a key mechanism of cardiomyocyte death after AMI.
• Gemfibrozil attenuates caspase-11 activation during ischemia/reperfusion in mice.
• Gemfibrozil reduces myocardial infarct size and prevents necrotic cell death.
• This study suggests gemfibrozil as a potential therapy to limit AMI damage.

## Graphical abstract

**Figure:**
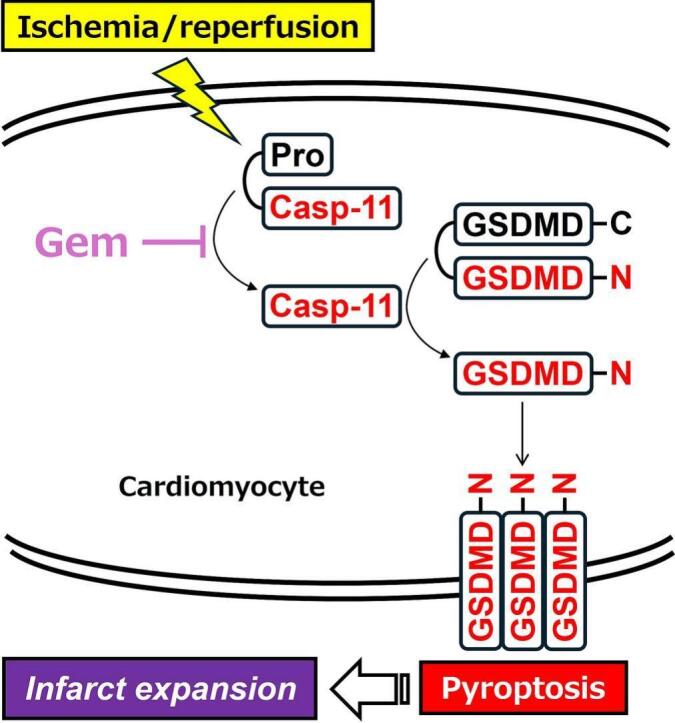

## 1 Introduction

Acute myocardial infarction (AMI) is a leading cause of sudden death and significant contributor to both acute and chronic heart failure. The size of the infarct area is a critical determinant of prognosis following AMI. However, the mechanisms underlying myocardial cell death after AMI are poorly understood. Traditionally, apoptosis and necrosis-like cell death have been implicated in myocardial cell death after AMI [1,2]. Necrosis-like cell death, characterized by the disruption of cell membrane integrity due to excessive influx and efflux of substances [1], encompasses various forms such as pyroptosis, necroptosis, and ferroptosis [3]. Among these, pyroptosis has recently gained attention as a significant form of cell death following AMI [4]. Pyroptosis is mediated by intracellular signaling pathways, with the NLR family pyrin domain-containing protein 3 (NLRP3) inflammasome playing a central role [5]. This canonical pyroptosis pathway involves caspase-1 [6], which undergoes autocleavage upon activation by the NLRP3 inflammasome. Activated caspase-1 cleaves gasdermin D (GSDMD), the primary executor of pyroptosis [6]. The N-terminal fragment of GSDMD (GSDMD-N) oligomerizes to form pores in the cell membrane, disrupting its integrity and triggering cell death [6]. The NLRP3 inflammasome is activated by damage-associated molecular patterns (DAMPs) and/or decreases in intracellular potassium (K^+^) levels [6]. Recent studies have also highlighted the involvement of caspase-11-induced non-canonical pyroptosis pathway in cardiomyocyte death after AMI [7]. Caspase-11, activated through self-cleavage, cleaves GSDMD and initiates pyroptosis [8]. Furthermore, cleaved caspase-11 can activate caspase-1 [9], contributing to the NLRP3 inflammasome signaling pathway. Considering its role in these pathways, caspase-11 represents a promising therapeutic target for preventing myocardial cell death after AMI. However, no drugs have been developed to inhibit myocardial cell death through caspase-11 inhibition.

Gemfibrozil (Gem), a lipid-lowering drug, has been suggested to directly inhibit caspase-11 in addition to its established role in stimulating peroxisome proliferator-activated receptor α (PPARα) [10]. Fibrates, including Gem, are reported to have cardioprotective effects after AMI [[11], [12], [13]], although the underlying mechanisms remain unclear. Furthermore, the effects of Gem on myocardial cell death following AMI have not been explored.

This study aimed to investigate the effects of Gem on caspase-11-dependent pyroptosis in the myocardial infarct area after AMI. To this end, ischemia/reperfusion (I/R) experiments were performed in mice, and primary cultured cardiomyocytes isolated from adult mice were subjected to hypoxia/reoxygenation.

## 2 Materials and methods

### 2.1 Animals

Ten-week-old male C57Bl/6n mice (SLC, Hamamatsu, Japan) were used in the present study. The mice were maintained at 23 ± 1 °C in an animal room with a constant humidity of 55 ± 5 %, a 12-h light/dark cycle, and free access to food and tap water according to the Guide for the Care and Use of Laboratory Animals published by the US National Institutes of Health (NIH Publication No. 85-23, revised 2011). The protocol of the present study was approved by the Animal Care and Use Committee of Tokyo University of Pharmacy and Life Sciences (P24–03).

### 2.2 Preparation of myocardial I/R mice

Isoflurane (induction: 5 %, maintenance: 3 %, Pfizer, New York, NY, USA) was used for anesthesia during open-chest surgery, and the breathing of the mice was maintained with a small-animal ventilator (MiniVent Ventilator for Mice, Model 845, Harvard Apparatus, Holliston, MA, USA). AMI was induced by ligation of the left coronary artery at a position 4 mm from the apex of the heart using a 7–0 silk suture thread (Medtronic plc, Dublin, Ireland) together with a 25G needle-made spacer. One hour after ligation, the spacer was removed, and blood reperfusion was confirmed. After reperfusion, the hearts were removed at 0, 3, 6, 12, 24, or 48 h and used for various experiments. Gem (Tokyo Chemical Industry Co., Ltd., Tokyo, Japan) was administered intraperitoneally at doses of 10, 30, and 100 mg/kg, twice in total, immediately after reperfusion and 24 h after reperfusion.

### 2.3 Experimental groups

We used 120 mice in the present study. A total of 6 mice were used in each of the 7 groups: 0, 3, 6, 12, 24, and 48 h after reperfusion, and the control group. In addition, 6 mice were used in each of the 5 groups of I/R with Gem administered at 0, 10, 30, and 100 mg/kg and Sham. For Evans blue/2,3,5-triphenyltetrazolium chloride (TTC) staining and immunostaining, 12 mice were used in each of the 4 groups of I/R for 48 h and Sham with or without 30 mg/kg Gem administration. In addition, 10 mice were used for the isolation and culture of cardiomyocytes.

### 2.4 Creatine kinase (CK)-MB assay

The measurement of CK-MB activity was performed as previously described [14]. The mice were heparinized (1 U/g, Nipro Corp., Osaka, Japan) and anesthetized with isoflurane (induction: 5 %, maintenance: 3 %), and then blood was collected from the right ventricle. The collected blood was centrifuged to separate the serum. (1200 ×g, 4 °C, 5 min). The L-type Wako CK-MB Kit (FUJIFILM Wako Pure Chemical Corporation, Osaka, Japan) was used to measure CK-MB activity.

### 2.5 Evans Blue/TTC staining

Forty-eight hours after I/R or Sham surgery, the mice were anesthetized with isoflurane (induction: 5 %, maintenance: 3 %), and the breathing of the mice was maintained with a small-animal ventilator. The left coronary artery was then ligated again with a 7–0 silk suture, and 5 % Evans Blue solution (Tokyo Chemical Industry Co., Ltd.) was administered *via* the abdominal vena cava. After the heart was quickly removed, the excess EB solution was washed off with saline. Hearts were sliced at 1 mm intervals and immersed in 1 % TTC solution (FUJIFILM Wako Pure Chemical Corporation) for 10 min (37 °C).

### 2.6 Echocardiographic measurements

Forty-eight hours after I/R or Sham operation, the mice were anesthetized with isoflurane (2 %), and then measured echocardiography as described previously [15]. Two-dimensional and Doppler imaging were performed by using an ARIETTA 70 (Hitachi-Aloka, Tokyo, Japan) equipped with high-frequency linear probe (5–18 MHz, L64 linear probe, Hitachi-Aloka). The left ventricular internal diameters at end diastole (LVIDd) and systole (LVIDs) were measured from M-mode tracings. Then, the left ventricular fractional shortening (FS), a left ventricular systolic function, was calculated by using these left ventricular internal diameters at end diastole and systole.

### 2.7 Isolation and culture of adult cardiomyocytes

The isolation procedure of cardiomyocytes from adult mice was performed as previously described [14]. Isolated cardiomyocytes were suspended in culture medium (M199 (Sigma-Aldrich, St. Louis, MO, USA) containing 10 % FBS (Biowest, Nuaillé, France) and 10 mmol/L 2,3-butandione 2-monoxime (FUJIFILM Wako Pure Chemical Corporation)) and then seeded onto laminin (FUJIFILM Wako Pure Chemical Corporation)-coated 35-mm dishes or 4-well glass slides. One hour after seeding, the medium was changed to remove unattached cells, and the cells were cultured for a further 24 h. Anaero Pack-Kenki (Mitsubishi Gas Chemical Co., Inc., Tokyo, Japan), an oxygen absorber and carbon dioxide generator, was used for hypoxic culture. The culture medium was replaced with a hypoxic buffer (120 mmol/L NaCl, 21 mmol/L HEPES (pH 7.4), 11 mmol/L mannitol, 4.8 mmol/L KCl, 1.2 mmol/L KH_2_PO_4_, 1.2 mmol/L MgSO_4_ 7H_2_O, 1 mmol/L CaCl_2_ 2H_2_O (Nacalai Tesque, Inc., Kyoto, Japan)) and the cultured cardiomyocytes were placed in a jar with an Anaero Pack-Kenki, which was then sealed and incubated in a 37 °C incubator. After 2 h, the hypoxic cultured cardiomyocytes were removed from the jar and reoxygenated by replacing the culture medium. The hypoxic/reoxygenated cardiomyocytes were used for various experiments 6 h after the start of reoxygenation.

### 2.8 Molecular docking simulation of gemfibrozil with caspase-11

AutoDock Vina 1.2.3. [16,17] was used for molecular docking simulations as previously described [18]. The caspase-4 (caspase-11)/pro-IL-18 complex (8SPB) provided by the Protein Data Bank (PBD), removed with pro-IL-18, was used as a three-dimensional model of caspase-11. The three-dimensional model of Gem was drawn in ChemDraw 20.0 (PerkinElmer, Inc., Waltham, MA, USA). Molecular docking models were drawn using the molecular graphics tool PyMOL (Schrödinger, Inc., New York, NY, USA).

### 2.9 Western blotting and detection of proteins

Sample preparation for western blotting was performed according to the method described previously [18]. Mice were anesthetized with isoflurane, and then their hearts were isolated and divided into ischemic area and viable area of left ventricular walls. Cultured cells were scraped 6 h after the start of reoxygenation. The collected cells were centrifuged (1000 ×*g*, 4 °C, 10 min), and the supernatant was removed. Isolated cardiac tissues and cultured cells were frozen in liquid nitrogen and stored at −80 °C prior to use.

Western blot analysis was performed according to the method described earlier [19]. In the present study, chemiluminescence quantification was performed by using ECL prime or ECL select (Cytiva, Tokyo, Japan), followed by image acquisition with a Fusion SOLO 6S EDGE (Vilber Lourmat, Marne-la-Vallée, France). The following antibodies were used for the samples of mouse heart: anti-caspase-11 (#14340, 1:1000, Cell Signaling Technology, Inc., Danvers, MA, USA), anti-GSDMD (#39754, 1:1000, Cell Signaling Technology, Inc.), and anti-actin (A4700, 1:10000, Sigma-Aldrich) antibodies.

### 2.10 Immunostaining

The method for immunostaining was described previously [19]. Anti-GSDMD (#39754, Cell Signaling Technology, Inc.) was used for primary antibody, and anti-rabbit IgG DyLight 549 (Vector Laboratories, Newark, CA, USA) was used for secondary antibody. For staining of sarcolemma, fluorescein (FITC)-conjugated wheat germ agglutinin (WGA) (Vector Laboratories) was used. The stained cardiomyocytes were observed using an all-in-one fluorescence microscope (BZ-X800, KEYENCE, Osaka, Japan) equipped with an optical sectioning module (BZ-H4XF, KEYENCE).

### 2.11 Cell death assay in cultured cardiomyocytes

The necrotic cell death assay was performed using an Apoptotic, Necrotic, and Healthy Cells Quantification Kit (Biotium, Inc., Fremont, CA, USA). Cells were stained 6 h after reoxygenation. The stained cells were observed with a microscope digital camera (DP80, Olympus, Tokyo, Japan) as described above [14,19].

### 2.12 Lactate dehydrogenase assay in cultured cardiomyocytes

The lactate dehydrogenase (LDH) assay was performed using a Cytotoxicity LDH Assay Kit-WAS (Dojindo Lab., Inc., Kumamoto, Japan). Cultured supernatant after 6 h of reoxygenation was used. The absorbance was measured using a microplate reader (MTP-310, Corona Electric Co., Ltd., Ibaraki, Japan).

### 2.13 Statistics

The experimental results were expressed as mean ± standard deviation (SD). JMP Pro 17 software (JMP Statistical Discovery LLC., Cary, NC, USA) was used for statistical analyses. The experimental data were analyzed using Tukey's and Dunnett's method, with *p* < 0.05 considered a statistically significant difference.

## 3 Results

### 3.1 Changes in cleaved caspase-11 and GSDMD levels in the ischemic region of ischemic/reperfused hearts

**Fig. 1:**
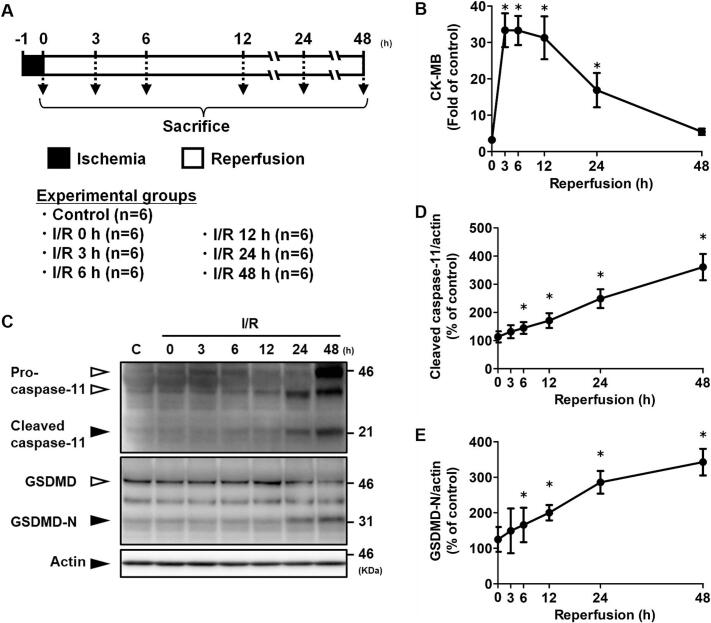
Cleaved caspase-11 and GSDMD-N levels in the ischemic region at 0, 3, 6, 12, 24, and 48 h after reperfusion. (A) Scheme of the experimental design. (B) CK-MB activity. (C) Representative western blot images of caspase-11 and GSDMD. (D) Quantification of cleaved caspase-11 levels. (E) Quantification of GSDMD-N levels. Data are presented as the mean ± standard deviation of six animals. The experimental data were analyzed using Dunnett's method. *p < 0.05 *vs.* the control group.

The levels of cleaved caspase-11 and GSDMD in the ischemic region of cardiac tissue were measured at 0, 3, 6, 12, 24, and 48 h after reperfusion (Fig. 1). Cleaved caspase-11, an indicator of caspase-11 activation, began to increase at 6 h post-reperfusion (Fig. 1C, D). Similarly, the active form of GSDMD, GSDMD-N, showed a marked increase starting at 6 h after reperfusion (Fig. 1C, E).

### 3.2 Gem inhibits activation of caspase-11 and GSDMD in the ischemic region of ischemic/reperfused hearts

**Fig. 2:**
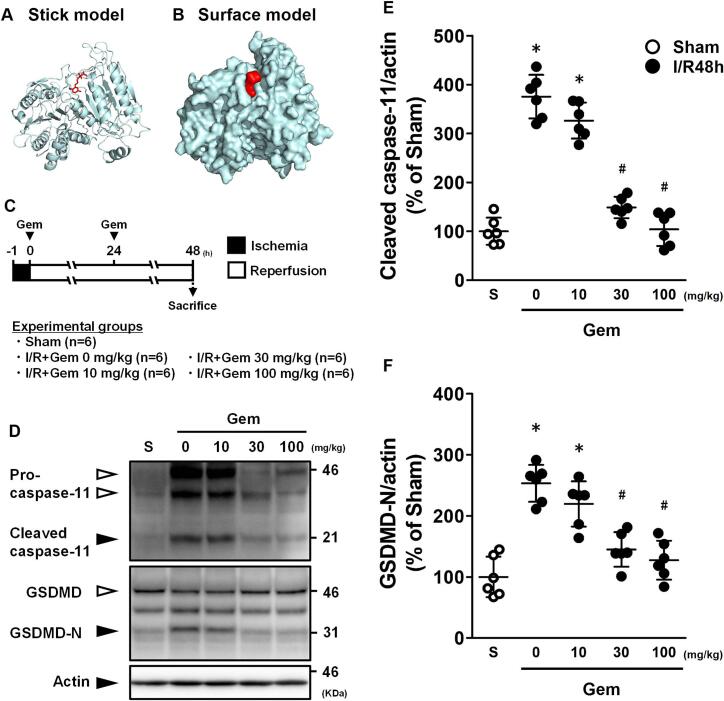
Effects of gemfibrozil (Gem) on caspase-11 and GSDMD levels in the ischemic region of mice 48 h after ischemia/reperfusion (I/R) (black plots) and Sham (white plots). (A) Stick model and (B) surface model from molecular docking simulations showing Gem binding to caspase-11. (C) Scheme of the experimental design. (D) Representative western blot images of caspase-11 and GSDMD. (E) Quantification of cleaved caspase-11 levels. (F) Quantification of GSDMD-N levels. Data are presented as the mean ± standard deviation of six animals. The experimental data were analyzed using Tukey's method. *p < 0.05 *vs.* the Sham group. #*p* < 0.05 *vs.* the vehicle-treated (0 μmol/L) group 48 h after I/R (IR48h).

Docking simulations revealed potential binding of Gem to caspase-11, with an affinity of −7.31 kcal/mol, suggesting its ability to bind near the ligand-binding site of caspase-11 (Fig. 2A, B).

The levels of cleaved caspase-11 and GSDMD-N in ischemic myocardial tissue were significantly reduced in Gem-treated groups compared to untreated controls. Both the 30 and 100 mg/kg doses of Gem substantially decreased cleaved caspase-11 levels (Fig. 2E) and GSDMD-N levels (Fig. 2F).

### 3.3 Gem reduces myocardial infarction area and improves cardiac function

**Fig. 3:**
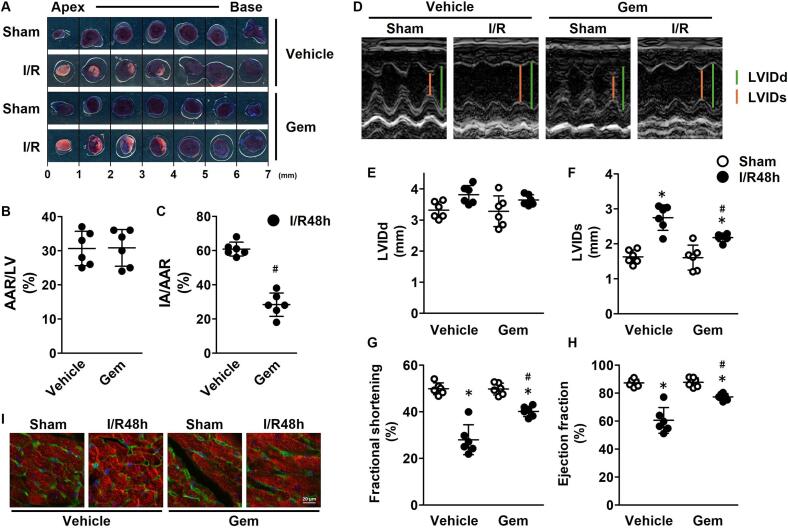
Effects of gemfibrozil (Gem) on infarct size and cardiac function 48 h after ischemia/reperfusion (I/R) (black plots) and Sham (white plots). (A) Representative Evans Blue (EB)/TTC-stained images showing infarct areas. (B) AAR/LV ratio (area at risk as a percentage of the left ventricle). (C) IA/AAR ratio (infarct area as a percentage of the area at risk). (D) Representative M-mode echocardiographic images. (E) Left ventricular internal diameter in diastole (LVIDd). (F) Left ventricular internal diameter in systole (LVIDs). (G) Fractional shortening. (H) Ejection fraction. (I) Representative immunohistochemical images showing GSDMD (red), FITC-WGA (green), and nuclei (blue) in ischemic regions. Data are presented as the mean ± standard deviations of six animals. The experimental data were analyzed using Tukey's method. *p < 0.05 *vs.* the Sham group. #*p* < 0.05 *vs.* the vehicle-treated (0 μmol/L) group 48 h after I/R (IR48h). (For interpretation of the references to colour in this figure legend, the reader is referred to the web version of this article.)

The impact of Gem on myocardial infarct size was assessed 48 h after reperfusion. In the vehicle-treated group, the area at risk (AAR) for myocardial infarction, identified as the Evans Blue-unstained area following left coronary artery ligation, constituted approximately 30 % of the left ventricle (LV; Fig. 3B). The infarct area (IA), defined as the TTC-unstained portion within the AAR, accounted for approximately 60 % of AAR in the vehicle group. Although the AAR/LV ratio was similar between vehicle and Gem-treated groups, the IA/AAR ratio (Fig. 3C) was significantly reduced in the Gem-treated group, indicating a smaller infarct size.

Echocardiographic analysis revealed that reperfusion injury in the vehicle group led to impaired cardiac function, evidenced by a decrease in fractional shortening (Fig. 3G) and ejection fraction (Fig. 3H), accompanied by an increase in left ventricular internal dimension at systole (LVIDs; Fig. 3F). Treatment with Gem (30 mg/kg) mitigated these changes, preventing the decline in fractional shortening and ejection fraction by attenuating the increase in LVIDs. These findings indicate that Gem improves cardiac function by reducing infarct size after I/R.

Immunohistochemical analysis (Fig. 3I) showed accumulation of GSDMD on the membranes of myocardial cells in tissue sections 48 h after reperfusion. Treatment with Gem effectively prevented this accumulation.

### 3.4 Effects of Gem on necrosis-like cell death in cultured adult mouse cardiomyocytes after hypoxia/reoxygenation

**Fig. 4:**
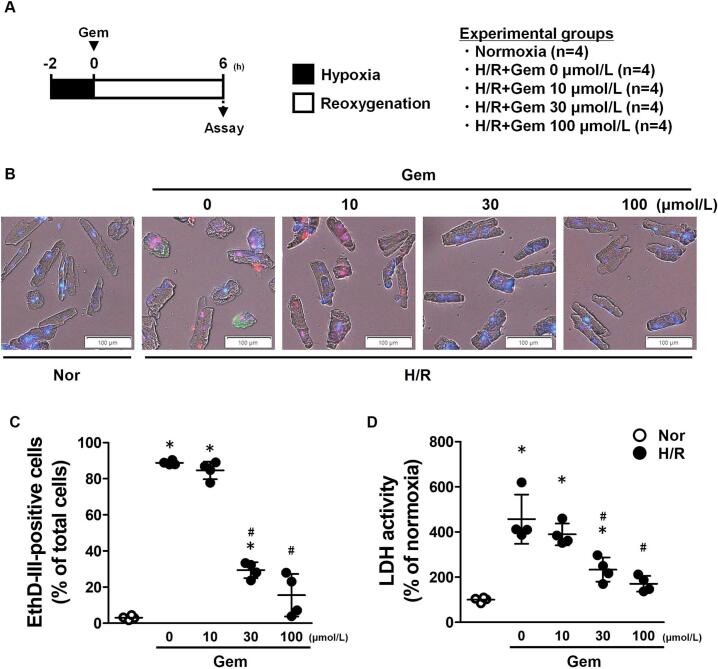
Effects of gemfibrozil (Gem) on hypoxia/reoxygenation (H/R)-induced necrotic cell death in cultured adult mouse cardiomyocytes. (A) Scheme of the experimental design. (B) Representative images of cardiomyocytes stained with ethidium homodimer III (EthD-III, red), annexin V (green), and Hoechst 33342 (blue). (C) Percentage of EthD-III-labeled necrotic cells among total cells. (D) Lactate dehydrogenase (LDH) activity in cultured supernatants. Data are presented as the mean ± standard deviations of four independent experiments. The experimental data were analyzed using Tukey's method. *p < 0.05 *vs.* the Normoxia (Nor, white plot) group. #*p* < 0.05 *vs.* the vehicle-treated (0 μmol/L) H/R group. (For interpretation of the references to colour in this figure legend, the reader is referred to the web version of this article.)

Representative images of cell death staining in cultured cardiomyocytes 6 h after reoxygenation, with or without Gem treatment, are shown in Fig. 4B. The percentage of necrosis-like cell death, indicated by ethidium homodimer III-positive staining (Fig. 4C), was approximately 90 % in the untreated group (0 μmol/L). Similar results were observed in the 10 μmol/L group, whereas necrosis-like cell death was markedly reduced to approximately 30 % in the 30 μmol/L group and 15 % in the 100 μmol/L group. LDH activity in the culture supernatant was also measured as a marker of cell death (Fig. 4D). In the 0 μmol/L group, LDH activity increased to approximately 4.5 times that of the normoxia group. The 10 μmol/L group exhibited comparable LDH activity to the 0 μmol/L group. However, LDH activity was significantly reduced in the 30 μmol/L and 100 μmol/L Gem-treated groups, measuring approximately 2.3 and 1.7 times the normoxia levels, respectively. These findings indicate that Gem effectively reduces necrosis-like cell death in cultured cardiomyocytes after hypoxia/reoxygenation.

### 3.5 Gem inhibits activation of caspase-11 and GSDMD in cultured adult mouse cardiomyocytes after hypoxia/reoxygenation

**Fig. 5:**
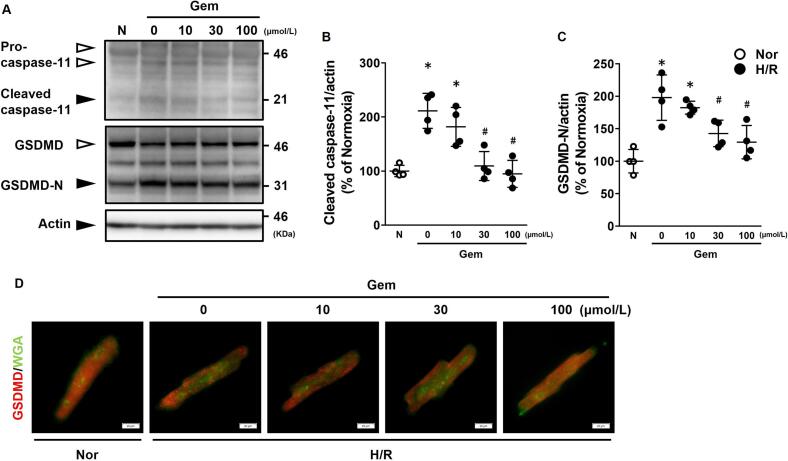
Effects of gemfibrozil (Gem) on caspase-11 and GSDMD levels in cultured adult mouse cardiomyocytes after hypoxia/reoxygenation (H/R). (A) Representative western blot images of caspase-11 and GSDMD. (B) Quantification of cleaved caspase-11 levels. (C) Quantification of GSDMD-N levels. (D) Representative immunohistochemical images showing GSDMD (red) and FITC-WGA (green) in cultured cardiomyocytes. Data are presented as the mean ± standard deviation of four independent experiments. The experimental data were analyzed using Tukey's method. *p < 0.05 *vs.* the Normoxia (Nor, white plot) group. #*p* < 0.05 *vs.* the vehicle-treated (0 μmol/L) H/R group. (For interpretation of the references to colour in this figure legend, the reader is referred to the web version of this article.)

The levels of cleaved caspase-11 and GSDMD-N in cultured adult mouse cardiomyocytes after hypoxia/reoxygenation were analyzed with or without Gem treatment (Fig. 5A–5C). Cleaved caspase-11 levels (Fig. 5B) and GSDMD-N levels (Fig. 5C) were significantly lower in the 30 and 100 μmol/L Gem-treated groups compared to the 0 μmol/L group, indicating effective inhibition of pyroptosis by Gem. Immunohistochemical analysis further supported these results (Fig. 5D). In the untreated group, GSDMD accumulated on the cell membranes of cultured cardiomyocytes 6 h after reoxygenation. However, Gem treatment prevented the membrane accumulation of GSDMD.

## 4 Discussion

Human caspase-4 has been identified as the functional ortholog of murine caspase-11 [20], and molecular docking simulations were conducted using the human caspase-4 model (PDB ID: 8SPB) with Gem. Caspase-11, as an upstream regulator in the caspase-1/GSDMD pathway [8,9], is believed to play a critical role in GSDMD-dependent pyroptosis. While caspase-1-mediated cell death, regulated by the NLRP3 inflammasome, has been extensively studied in the context of myocardial cell death after AMI, the role of caspase-11 in this process remains poorly understood.

NLRP3 activation is triggered by DAMPs such as ATP, high-mobility group box 1, mitochondrial DNA [21], and intracellular K^+^ depletion [22]. In contrast, caspase-11 serves as an intracellular receptor for lipopolysaccharides [23,24]. Recent research suggests that caspase-11 activation is also mediated by reactive oxygen species (ROS) [25], which are produced in excess during myocardial I/R [26]. This suggests that ROS may enhance caspase-11 activation, contributing to myocardial cell death following AMI. Supporting this hypothesis, studies have demonstrated that the exposure of cultured cardiomyocytes to hydrogen peroxide induces caspase-11 activation [7]. Thus, it is postulated that caspase-11 is activated by excessive ROS production during reperfusion, rather than ischemia, leading to the development of reperfusion injury.

Our study showed that caspase-11 activation begins 6 h after reperfusion on-set, representing a relatively late phase in acute myocardial cell death, and persists for up to 48 h post-reperfusion. These findings indicate that targeting this pathway could effectively prevent cell death during the late stages of reperfusion.

Currently available caspase-11 inhibitors are peptide-based compounds. Smith et al. [10] conducted a high-throughput screen for caspase-4 inhibitors and identified several non-steroidal anti-inflammatory drugs (NSAIDs) with inhibitory effects on caspase-11. However, NSAIDs have demonstrated no beneficial effects on AMI or subsequent heart failure [[27], [28], [29]]. In this study, we focused on Gem as a potential inhibitor of caspase-11 from the database presented by Smith et al. [10]. Our molecular docking simulations (Fig. 2A and B) revealed that Gem has a favorable binding affinity to caspase-11. Notably, no prior studies have evaluated the effects of Gem on AMI or heart failure. Our findings indicate that the timing of Gem administration is crucial, as it was most effective when administered at the onset of reperfusion and 24 h post-reperfusion, coinciding with the timeline of caspase-11-dependent pyroptosis (6–48 h post-reperfusion). This suggests that Gem could be a viable treatment option for AMI.

Gem is a well-known PPARα agonist. Several investigators showed that pretreatment with selective PPARα agonists GW7647 and WY-14643 protected myocardium against I/R injury in rodents [30,31]. Furthermore, treatment of rats with fenofibrate mitigated myocardial I/R injury during 2 h reperfusion *via* a reduction in myocardial endoplasmic reticulum stress [32], In contrast, clinical studies using fibrates reported no significant impact on cardiovascular mortality [33,34]. There are no clinical studies that evaluate specifically cardioprotective effects of Gem alone in AMI. Our findings suggest, at least in part, that protective effects of Gem on cardiac function and metabolism against I/R injury are attributable to an inhibition of caspase-11. The present study, conducted at a basic research level using animal models, provides novel insights into the therapeutic potential of Gem for AMI. Future investigations, including clinical trials, are warranted to further explore its role in myocardial ischemic diseases. Our findings may contribute to the development of new treatment strategies for AMI.

## 5 Conclusions

This study demonstrated that Gem treatment significantly reduced infarct size in I/R mice. This effect appears to be mediated, at least in part, by the inhibition of caspase-11-mediated GSDMD activation. These findings suggest that therapeutic administration of Gem or other caspase-11 inhibitors could represent a promising novel approach for the treatment of AMI.

## CRediT authorship contribution statement

**Tetsuro Marunouchi:** Writing – original draft, Visualization, Validation, Software, Methodology, Investigation, Funding acquisition, Formal analysis, Data curation, Conceptualization. **Mayu Kyono:** Investigation. **Naoko Kikuchi:** Investigation. **Kouichi Tanonaka:** Writing – review & editing, Validation, Supervision, Project administration, Methodology, Funding acquisition, Conceptualization.

## Ethical approval

All procedures involving animals adhered to applicable International, National, and Institutional Guidelines for the Care and Use of Animals. The study protocol was approved by *the Committee of Animal Use and Welfare of Tokyo University of Pharmacy and Life Sciences* (Approval Number: P24-02).

## Declaration of Generative AI and AI-assisted technologies in the writing process

No generative AI or AI-assisted technologies were used in the preparation of this article, except for grammar and spelling checks.

## Funding

This work was supported by the 10.13039/501100001691JSPS KAKENHI Grant (Grant Number: JP22K15297).

## Declaration of competing interest

The authors declare no competing interests.
